# *Lactobacillus plantarum* 299V improves the microbiological quality of legume sprouts and effectively survives in these carriers during cold storage and *in vitro* digestion

**DOI:** 10.1371/journal.pone.0207793

**Published:** 2018-11-21

**Authors:** Michał Świeca, Monika Kordowska-Wiater, Monika Pytka, Urszula Gawlik-Dziki, Justyna Bochnak, Urszula Złotek, Barbara Baraniak

**Affiliations:** 1 Department of Biochemistry and Food Chemistry, University of Life Sciences in Lublin, Lublin, Poland; 2 Department of Biotechnology, Microbiology and Human Nutrition, University of Life Sciences in Lublin, Lublin, Poland; University of Torino, ITALY

## Abstract

Probiotics improve consumers' health and additionally may positively influence the microbiological and organoleptic quality of food. In the study, legume sprouts were inoculated with *Lactobacilllus plantarum* 299V to produce a new functional product ensuring the growth and survival of the probiotic and high microbiological quality of the final product. Legume sprouts, which are an excellent source of nutrients, were proposed as alternative carriers for the probiotic. The key factors influencing the production of probiotic-rich sprouts include the temperature (25°C) of sprouting and methods of inoculation (soaking seeds in a suspension of probiotics). Compared to the control sprouts, the sprouts enriched with the probiotic were characterized by lower mesophilic bacterial counts. In the case of fresh and stored probiotic-rich sprouts, lactic acid bacteria (LAB) accounted for a majority of total microorganisms. The *Lb*. *plantarum* population was also stable during the cold storage. The high count of LAB observed in the digest confirmed the fact that the studied sprouts are effective carriers for probiotics and ensure their survival in the harmful conditions of the digestive tract in an *in vitro* model. Enrichment of legume sprouts with probiotics is a successful attempt and yields products for a new branch of functional foods.

## Introduction

Probiotics are “live strains of strictly selected microorganisms which, when administered in adequate amounts, confer a health benefit on the host” [1], [2]. In nature, *Lactobacillus plantarum* is found in a variety of ecological niches, ranging from vegetables and fermented food (e.g. vegetables, cheeses, meat) to the human gastrointestinal tract [3], [4]. *Lb*. *plantarum* 299V is widely used as a single probiotic or in synbiotic formulations and exhibits a wide range of positive effects on human health. The effectiveness of *Lb*. *plantarum* 299V for treatment of gastrointestinal diseases (e.g., inflammatory bowel disease, diarrheas), allergic diseases (e.g., atopic dermatitis), obesity, insulin resistance syndrome, type 2 diabetes, and nonalcoholic fatty liver disease has been confirmed by many clinical studies [5], [6], [7]. Furthermore, probiotic strains of *Lb*. *plantarum* are also responsible for immunomodulation (increasing human immunity), and can thus be used as a prophylactic agent and/or cure for autoimmune disorders, inflammation, and different types of cancers and side effects associated with cancer [8], [9].

Probiotics are usually delivered as food supplements in the form of tablets or capsules [1]. In such formulations, they are usually combined with prebiotics, which are selective substrates for one or a limited number of probiotics [5], [10]. On the other hand, probiotics can also positively influence the microbiological and organoleptic quality of food; thus, they are widely incorporated into traditional food products, e.g., dairy products, fermented vegetables, and meats [3], [11], [12]. In recent years, some alternative matrices, e.g., chocolate, fruit drink, and cut fruits have been employed as carriers for these beneficial microorganisms [13], [14]. This need for alternative matrices is related to the new diet habits, e.g., veganism and/or the higher number of consumers with diet restrictions such as those with lactose intolerance, allergies, and cholesterol restriction.

Legumes are an excellent source of nutrients (including resistant starch, which can make them effective prebiotics) and components with well-documented pro-health properties, e.g., fiber, phenolics, or vitamins [15], [16]. Additionally, the quality of pulses can be improved by sprouting, i.e. a process effectively increasing nutrient digestibility, bioavailability of minerals, and the content of low-molecular weight antioxidants [17], [18]. Sprouted food is susceptible to microbiological invasion due to the presence of simple nutrients (free sugars, vitamins, and amino acids), high content of water, and the fact that seeds used for germination cannot be sterilized [19], [20]. To preserve the shelf life of sprouts, many techniques are employed including chemical (limited by formation of by-products, which could have implications for human health) [21] and physical treatments [22].

A new functional food based on legume sprouts and *Lb*. *plantarum* was designed for the first time. It was assumed that such a combination ensures both the growth and survival of the probiotic and high microbiological quality of the final product. The factors affecting production of probiotic-rich sprouts, including selection of legume species, conditions of germination, and methods for inoculation with the probiotics, were optimized in the screening studies. For the selected preparations, microbiological quality (degree of contamination) and the *Lb*. *plantarum* survival efficiency during cold storage and *in vitro* digestion were determined.

## Materials and methods

### Materials

All chemicals used for cultivation of sprouts and microbiological media were purchased from Sigma–Aldrich company (Poznan, Poland) and BTL Ltd. (Łodz, Poland). Soybean, lentil, adzuki bean, and mung bean seeds were purchased from PNOS S.A. in Ozarów Mazowiecki, Poland.

The *Lb*. *plantarum* 299V strain was isolated from a commercial probiotic preparation. The culture was tested for cell morphology; biochemical tests and 16S rRNA sequencing were performed as well. Bacterial stocks used for the inoculation were stored at –80°C in MRS broth with 20% (v/v) glycerol. Prior to inoculation, aerobic cultures on MRS agar were cultivated twice for 24 h at 37°C. Then, the colonies were sterilely collected from Petri dishes, suspended in water, and used for inoculation of the sprouts. For preparation of the inoculum, the bacterial concentration was estimated by optical density (OD) analysis at 600 nm using a spectrophotometer Smart Spec Plus (Bio-Rad, USA). A previously determined standard curve was used to determine the number of cells in the suspension at the level of 9.00 log_10_ CFU/mL on the basis of the OD value.

### Production of probiotic-rich sprouts

#### Sprouting conditions

Seeds were disinfected in 1% (v/v) sodium hypochlorite for 10 min, drained, and washed with distilled water until they reached neutral pH. Then, they were soaked in distilled water (C, control) or a *Lb*. *plantarum* 299V water suspension (1 × 10^8^ CFU per 1 g of seeds (8.00 log _10_ CFU/ g)) (S, soaked with the probiotic). Lentil, soybean, adzuki bean, and mung bean seeds were soaked for 4, 4, 6, and 8 h, respectively. The seeds (approximately 12 g per plate) were dark-germinated for 4 days in a growth chamber (SANYO MLR-350H) on Petri dishes (Ø 125 mm) lined with absorbent paper. Seedlings were sprayed daily with 5 mL of Milli-Q water (C, S) or 5 mL of water suspension of the probiotic only on the 1st day of cultivation (1 × 10^8^ CFU per 1 g of seeds (8.00 log _10_ CFU/ g)) (W, watered with the probiotic). Sprouting was carried out at 25°C, 30°C, and 35°C (25, 30, and 35, respectively) with relative humidity 90%. In the screening studies, the sprouts were manually collected after 4 days and analyzed for sprouting efficiency, biomass accumulation, and the number of viable lactic acid bacteria [23]. During the production of the probiotic-rich sprouts, 4-day-old sprouts were manually collected and immediately used for the test (fresh sprouts) or were stored in polypropylene boxes at 4°C for 7 days (stored sprouts).

### Assessment of probiotic production efficiency

#### Assessment of sprouting efficiency

The seed germination percentage was calculated using the following formula:

Seedgerminationpercent[%]=(Numberofgerminatedseeds/Totalnumberofseeds)×100%.

#### Biomass accumulation

Sprout biomass accumulation was expressed as the mass of 10 sprouts. For each variant of sprouting, at least 100 sprouts were weighted [24]

#### Counting of *Lb*. *plantarum* 299V viable cells

Once sprouting was completed, the samples were collected and the total number of lactic bacteria cells was determined. To this end, 1 g of the sprouts was gently homogenized with 9 mL of Ringer’s solution and shaken for 10 min (60 rpm). Then, serial decimal dilutions of the sprout samples were done and 0.1 mL aliquots were placed on MRS agar in triplicates and incubated aerobically at 37°C for 48 h. The characteristic colonies were counted and their numbers were calculated as CFU/g of the fresh weight of sprouts.

#### Efficiency factor

To sum up the final effect of the protocol proposed for the probiotic-rich sprouts, the efficiency factor was calculated [23]:

Efficiencyfactor=(Seedgerminationpercent/Massof10sprouts)×Amountoftheprobiotic

### Microbiological quality

The following microbiological analyses were performed in accordance with Polish or European standards.

#### Total mesophilic bacteria

The total number of mesophilic bacteria was determined with the plate technique on nutrient agar according to PN-EN ISO 4833–2:2013 [25].

#### Lactic acid bacteria

The number of lactic acid bacteria was determined with the plate technique on MRS agar according to PN-ISO 15214:2002 [26].

#### Molds and yeasts

The number of yeasts and molds was determined with the plate technique on agar with glucose, yeast extract, and chloramphenicol according to PN-ISO 21527–1:2009 [27]. Yeast and molds were differentiated according to the morphology of colonies.

#### Coliforms

Coliforms were determined with the plate method on VRBL medium according to PN-ISO 4832:2007 [28].

***Enterococci***

The number of enterococci was determined with the plate method on Slanetz-Bartley agar according to PN-A-86034-10:1993 [29].

***Staphylococcus aureus***

The number of *S*. *aureus* was determined using the Baird-Parker plate method according to PN-EN ISO 6888–1:2001 [30]. A confirmatory test was used to detect coagulase activity (Bactident Coagulase Test).

***Bacillus cereus***

The number of *B*. *cereus* was determined with the plate method on MYP medium according to PN-EN ISO 7932:2005 [31]; typical colonies were subjected to confirmatory tests.

***Listeria monocytogenes***

The presence of *L*. *monocytogenes* was determined by preincubation in half-Fraser broth, selective propagation in Fraser broth, and selection and identification of colonies on Palcam and Oxford media according to PN-EN ISO 11290–1:2017 [32]. In order to confirm the presence of these microorganisms, biochemical tests were performed using a Microbact Biochemical Identification Kit-Listeria Identification Kit 12L (Oxoid Ltd.).

***Salmonella* spp**.

The presence of *Salmonella* spp. was tested by preincubation in peptone-buffered water, selective propagation in Rappaport-Vasiliadis medium, and isolation and identification of colonies on BGA and XLD agars according to PN-EN ISO 6579–1:2017 [33]. Questionable colonies were subjected to RapID One System (Remel Europe Ltd.) biochemical tests.

***Yersinia enterocolitica***

The presence of *Y*. *enterocolitica* was determined by broth multiplication PSB and identification of colonies on CIN medium according to PN-EN ISO 10273:2017 [34]. In order to confirm their presence, biochemical tests using Rapid One System (Remel Europe Ltd.) were performed.

### Antimicrobial properties of *Lb*. *plantarum* against selected foodborne pathogens

The *Lb*. *plantarum* 299V strains were described for antagonistic activity using the well diffusion method. Sterile Petri dishes were poured with nutrient agar and inoculated with the culture of each indicator strain separately (concentration 6 log CFU/mL) to obtain the growth in the form of turf. For the study, common food-borne pathogens (*E*. *coli*, *S*. *aureus*, *B*. *cereus*, *S*. *enterica* sv. Enteritidis, *S*. *enterica* sv. Anatum, and *L*. *monocytogenes*) isolated from unprocessed food of plant origin were used. After solidification, wells with a 9.0-mm diameter were cut out and filled with 100 μL of the cultures of *Lb*. *plantarum*. The plates were incubated at 37°C for 48 h and the diameters of growth inhibition zones were measured. Antimicrobial activity (*x*) was calculated as follows: *x* = *D* − *d*, where *D* is the inhibition zone diameter and *d* is the well diameter. Antimicrobial activity (*x*) was characterized and classified based on the inhibition growth zone diameters and described as low (*x* < 4 mm diameter), medium (*x* = 4–8 mm), high (*x* = 8–12 mm), and very high (*x* > 12 mm) [11].

### *In vitro* digestion

*In vitro* digestion was performed as described previously [35]. For simulated mastication and gastrointestinal digestion, 2 g of fresh sprouts were homogenized in 1.0 mL PBS buffer and 1 mL of simulated salivary fluid [15.1 mmol/L KCl, 3.7 mmol/L KH_2_PO_4_, 13.6 mmol/L NaHCO_3_, 0.15 mmol/L MgCl_2_ (H_2_O)_6_, 0.06 mmol/L (NH_4_)_2_CO_3_, 1.5 mmol/L CaCl_2_, and α-amylase (75 U/mL)], and shaken for 10 min at 37°C. Next, the samples were adjusted to pH 3 with 6 M HCl, suspended in 4 mL of simulated gastric fluid [6.9 mmol/L KCl, 0.9 mmol/L KH_2_PO_4_, 25 mmol/L NaHCO_3_, 47.2 mmol/L NaCl, and 0.1 mmol/L MgCl_2_ (H_2_O)_6_, 0.5 mol/L (NH_4_)_2_CO_3_, 0.15 mmol/L CaCl_2_, and pepsin (2,000 U/mL)] and shaken for 120 min at 37°C. After simulated gastric digestion, the samples were adjusted to pH 7 with 1 M NaOH and suspended in 8 mL of simulated intestinal fluid [6.8 mmol/L KCl, 0.8 mmol/L KH_2_PO_4_, 85 mmol/L NaHCO_3_, 38.4 mmol/L NaCl, 0.33 mmol/L MgCl_2_ (H_2_O)_6_, 0.15 mmol/L CaCl_2,_ 10 mmol/L bile extract, and pancreatin (2,000 U/ mL)]. The prepared samples underwent *in vitro* intestinal digestion for 120 min. Then, serial decimal dilutions of the digest were done in Ringer`s solution and 0.1 ml of aliquots were spread on the MRS agar plate using a glass cell spreader. The plates were incubated aerobically at 30°C for 48 h. Characteristic colonies were counted and their numbers were calculated as CFU/g of fresh weight of the sprouts.

### Statistics

All experimental results are mean ± S.D. of three parallel experiments. One-way analysis of variance (ANOVA) and Tukey’s post hoc test were used to compare the groups (STATISTICA 13, StatSoft, Inc., Tulsa, USA). Differences were considered significant at *p* ≤ 0.05.

## Results and discussion

This study evaluated legume sprouts as carriers for *Lb*. *plantarum*. In the first step, screening studies were performed to define optimal conditions for subsequent growth of sprouts and probiotics (Table 1).

**Table 1:** Effect of sprouting temperature and inoculation methods on biomass accumulation, sprouting efficiency, and *Lb*. *plantarum* growth/survival.

	Cultivation conditions	Sprouts			
		Lentil	Soybean	Adzuki bean	Mung bean
Massof 10 sprouts[g]	C25	1.31±0.03a	2.53±0.11a	1.47±0.09ab	1.41±0.12b
	C30	1.43±0.09ab	2.51±0.22a	1.57±0.07b	1.45±0.11b
	C35	1.29±0.10ab	2.31±0.35a	1.46±0.02ab	1.35±0.10b
	P25S	1.52±0.04c	2.54±0.12a	1.54±0.07bc	1.42±0.10b
	P25W	1.56±0.04c	2.56±0.12a	1.62±0.07c	1.46±0.10b
	P30S	1.52±0.06c	2.47±0.11a	1.57±0.10abc	1.26±0.05ab
	P30W	1.56±0.04c	2.42±0.09a	1.57±0.03c	1.19±0.03a
	P35S	1.43±0.23abc	2.56±0.18a	1.46±0.16abc	1.29±0.15ab
	P35W	1.45±0.10abc	2.55±0.15a	1.61±0.14abc	1.29±0.07b
Sproutingefficiency[%]	C25	86.9±6.4cd	79.8±3.2c	88.8±2.2ab	94.0±1.6b
	C30	77.4±4.9c	78.2±3.2c	86.3±4.1a	94.7±1.6b
	C35	9.28±1.2a	69.2±7.7b	85.3±2.1a	89.6±6.8ab
	P25S	83.5±2.5c	74.3±4.4bc	90.6±2.2ab	94.7±0.2b
	P25W	83.5±3.3c	74.3±5.3bc	90.6±2.1ab	94.7±0.9b
	P30S	81.8±0.8c	80.5±1.3c	93.2±1.6b	94.9±0.5b
	P30W	89.3±0.5d	74.7±2.1b	89.2±1.2ab	94.7±4.6b
	P35S	8.37±1.3a	56.0±3.5a	85.3±2.7a	83.3±0.5a
	P35W	18.6±4.3b	69.3±2.9b	87.4±2.2a	84.5±2.7a
Number of LAB[log_10_ CFU/ g f.m.]	C25	0	0	0	0
	C30	0	0	0	0
	C35	0	0	0	0
	SDs-S	6.50c	6.74b	6.87b	6.67c
	SDs-W	4.92a	5.22a	5.17a	5.59a
	P25S	7.64f	8.16e	7.86d	8.05g
	P25W	5.38a	6.69b	5.42a	6.74c
	P30S	7.40e	7.57d	7.53c	8.15e
	P30W	6.00b	5.56a	5.20a	7.12d
	P35S	7.31d	7.28c	6.90b	7.70f
	P35W	6.43c	5.98a	4.00a	6.14b

Means (± SD) in the columns followed by different letters are significantly different (n = 9; *p*≤ 0.05).

SDs- seeds; C- control; P- sprouts enriched with *Lb*. *plantarum;* S- sprouts obtained by soaking the seeds in the probiotic; W- sprouts obtained by watering 1-day-old sprouts with the probiotic. 25, 30, 35- temperature of sprouting: 25°C, 30°C and 35°C, respectively. LAB–lactic acid bacteria; CFU–colony forming unit, f.m.–fresh mass.

**Fig 1:**
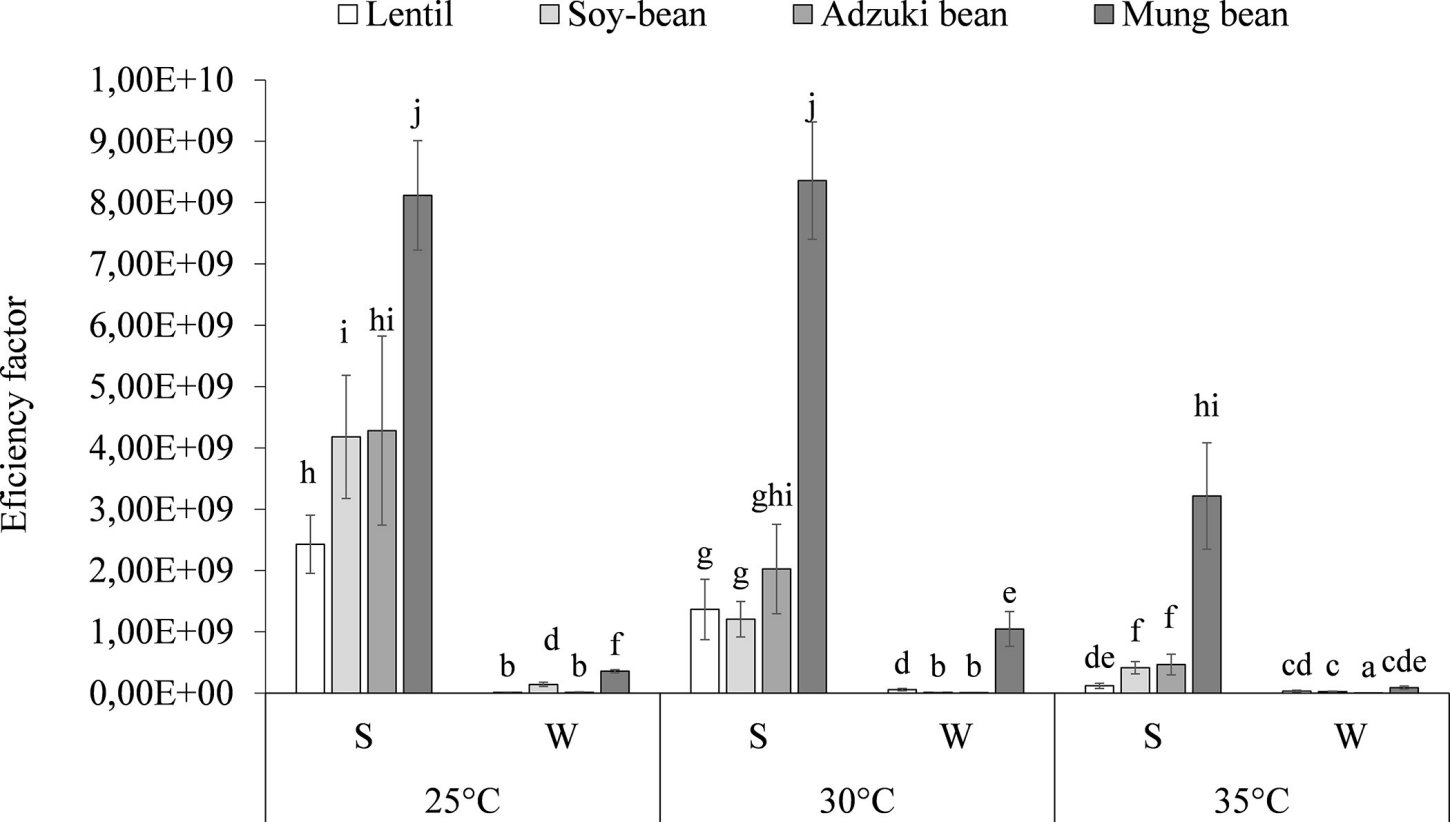
Effect of sprouting conditions and inoculation methods on the efficiency of production of probiotic-rich sprouts. S- sprouts obtained by soaking the seeds in the probiotic; W- sprouts obtained by watering 1-day-old sprouts with the probiotic. 25°C, 30°C, and 35°C- temperature of sprouting. Means followed by different letters are significantly different (n = 9; p≤ 0.05).

Generally, the beans (adzuki and mung) sprouted and grew effectively in the studied temperature range. The differences in the growth rate observed between the cultures did not exceed 5%. Most importantly, a significant reduction of the sprouting efficiency was observed at 35°C in the case of lentil and soybean. The largest count of lactic acid bacteria (LAB) was noted in the sprouts obtained at 25°C from seeds imbibed in the water suspension of *Lb*. *plantarum*. The LAB in the lentil, soybean, adzuki bean, and mung bean were 7.64, 8.16, 7.86, and 8.05 log_10_ CFU g^−1^ f.m., respectively (Table 1). The results of the germination rate, biomass accumulation, and the number of LAB were combined and efficiency factors were proposed (Fig 1).

This helped in determination of the conditions that are optimal for probiotic growth (survival) and sprout development. The best conditions for production of probiotic-rich sprouts, regardless of the type of legumes, were the same. The highest values of the efficiency factor were 2.43 × 10^9^, 4.18 × 10^9^, 4.28 × 10^9^, and 8.12 × 10^9^ for the preparations based on lentil, soybean, adzuki bean, and mung bean obtained at 25°C from seeds soaked in the *Lb*. *plantarum* water suspension, respectively (Fig 1). Following these results, preparations characterized by the highest value of the efficiency factor were selected for further detailed investigations to assess their microbiological quality and survival of the probiotic during *in vitro* digestion.

So far, legume sprouts were used as carriers for *Lb*. *rhamnosus* GG [23]. It was found that soybean sprouts inoculated by soaking the seeds in a water suspension of the bacteria were the best carrier. Similar to this study, inoculation with the imbibition step was more effective than spraying the 1-day-old sprouts. It may be suggested that bacteria absorbed into the seeds during soaking have access to higher amounts of nutrients necessary for their development and are protected against physical conditions, e.g., oxygen or drying (water activity). The germination temperature, inoculation methods, and legume species strongly influenced the efficiency of the production of probiotic-rich sprouts; a decrease in the sprouting efficiency was also observed in the case of soybean and lentil sprouting at 35°C. Intensive mobilization of storage materials and loosening of the seed structure [16] usually create favorable conditions for development of microbiota; thus, inhibition of sprouting observed at higher temperatures may limit probiotic growth and promote growth of pathogens and yeast able to utilize starch [36].

The results of the effect of *Lb*. *plantarum* on the microbiological quality of the control and probiotic-rich sprouts are presented in Table 2.

**Table 2:** Effect of *Lb*. *plantarum* on the microbiological quality of the control and probiotic-rich sprouts.

Count ofmicroorganisms[log_10_ CFU/ g f.m.]	Lentil				Soybean			
	Fresh		Stored		Fresh		Stored	
	C	P	C	P	C	P	C	P
Total mesophilic bacteria	8.71a	7.74a	9.91c	8.79b	8.77a	9.12a	9.63b	9.50b
Lactic bacteria	2.81a	7.61c	4.71b	8.45d	7.45a	9.06b	7.76a	9.29b
Molds	2.85a	2.60a	n.d.	1.91a	n.d.	n.d.	0.70	n.d.
Yeasts	n.d.	2.54a	n.d.	2.69a	n.d.	1.70a	n.d.	1.70a
Coliforms	n.d.	n.d.	6.14a	6.06a	n.d.	n.d.	8.46b	6.81a
*Enterococci*	n.d.	n.d.	n.d.	n.d.	2.24a	2.70b	2.30a	n.d.
*Staphylococcus aureus*	n.d.	n.d.	n.d.	n.d.	n.d.	n.d.	n.d.	n.d.
*Bacillus cereus*	2.00b	1.70a	2.12b	n.d.	2.73a	n.d.	3.18a	n.d.
*Listeria monocytogenes*	n.d.	n.d.	n.d.	n.d.	n.d.	n.d.	n.d.	n.d.
*Salmonella* spp.	n.d.	n.d.	n.d.	n.d.	n.d.	n.d.	n.d.	n.d.
*Yersinia enterocolitica*	n.d.	n.d.	n.d.	n.d.	n.d.	n.d.	n.d.	n.d.
Count ofmicroorganisms[log_10_ CFU/ g f.m.]	Adzuki bean				Mung bean			
	Fresh		Stored		Fresh		Stored	
	C	P	C	P	C	P	C	P
Total mesophilic bacteria	9.00b	8.43a	10.48d	10.13c	8.70a	8.78a	9.86b	9.90b
Lactic bacteria	2.61a	8.53c	3.83b	9.68d	5.97a	8.46b	5.42a	8.72b
Molds	n.d.	n.d.	1.70a	2.09b	n.d.	1.70a	n.d.	1.70a
Yeasts	n.d.	n.d.	n.d.	n.d.	n.d.	n.d.	n.d.	n.d.
Coliforms	n.d.	n.d.	6.80a	7.24b	n.d.	n.d.	7.72b	5.93a
*Enterococci*	n.d.	n.d.	n.d.	n.d.	3.16c	3.00b	2.88bc	2.18a
*Staphylococcus aureus*	n.d.	n.d.	n.d.	n.d.	n.d.	n.d.	n.d.	n.d.
*Bacillus cereus*	n.d.	1.70	n.d.	n.d.	2.18a	n.d.	2.00a	n.d.
*Listeria monocytogenes*	n.d.	n.d.	n.d.	n.d.	n.d.	n.d.	n.d.	n.d.
*Salmonella* spp.	n.d.	n.d.	n.d.	n.d.	n.d.	n.d.	n.d.	n.d.
*Yersinia enterocolitica*	n.d.	n.d.	n.d.	n.d.	n.d.	n.d.	n.d.	n.d.

Means in the rows (for the selected plants) followed by different letters are significantly different (n = 9; p≤ 0.05).

C- control; P- sprouts enriched with *Lb*. *plantarum;* CFU–colony forming unit. f.m.–fresh mass. n.d.- not detected.

Compared to the control sprouts, the lentil sprouts enriched with the probiotic were characterized by lower mesophilic bacterial counts (by about 89% and 92% in fresh and stored samples, respectively). Similar behavior was also observed in the fresh and stored probiotic-rich adzuki bean sprouts, stored soybean sprouts enriched with the probiotic, and fresh probiotic-rich mung bean sprouts. Only in the case of the fresh soybean and stored mung bean sprouts enriched with *Lb*. *plantarum*, the total count of mesophilic bacteria was higher than in the control counterparts. The lactic acid bacterial count was increased during storage of both the control and enriched sprouts in the all studied samples. In the control sprouts, their content did not exceed 1% of total microorganisms (except soybean sprouts). Most importantly, in the case of fresh and stored probiotic-rich soybean sprouts, LAB accounted for 86% of the total microorganisms. Similar growth was also observed in the preparations based on the lentil, adzuki, and mung bean sprouts. In all the studied samples, the numbers of yeast and molds were low or they were not detected. Coliforms were not present in the fresh samples but were detected after storage. No pathogenic *Listeria monocytogenes*, *Salmonella* spp., or *Y*. *enterocolitica* were found in any of the analyzed samples. Only in the case of the fresh and stored control soybean sprouts was there some evidence of the presence of *Listeria grayi*. Furthermore, *S*. *aureus* was not detected in the studied sprouts. Some incidental contamination with *Bacillus cereus* was also observed, especially in the fresh and stored control soybean sprouts.

**Fig 2:**
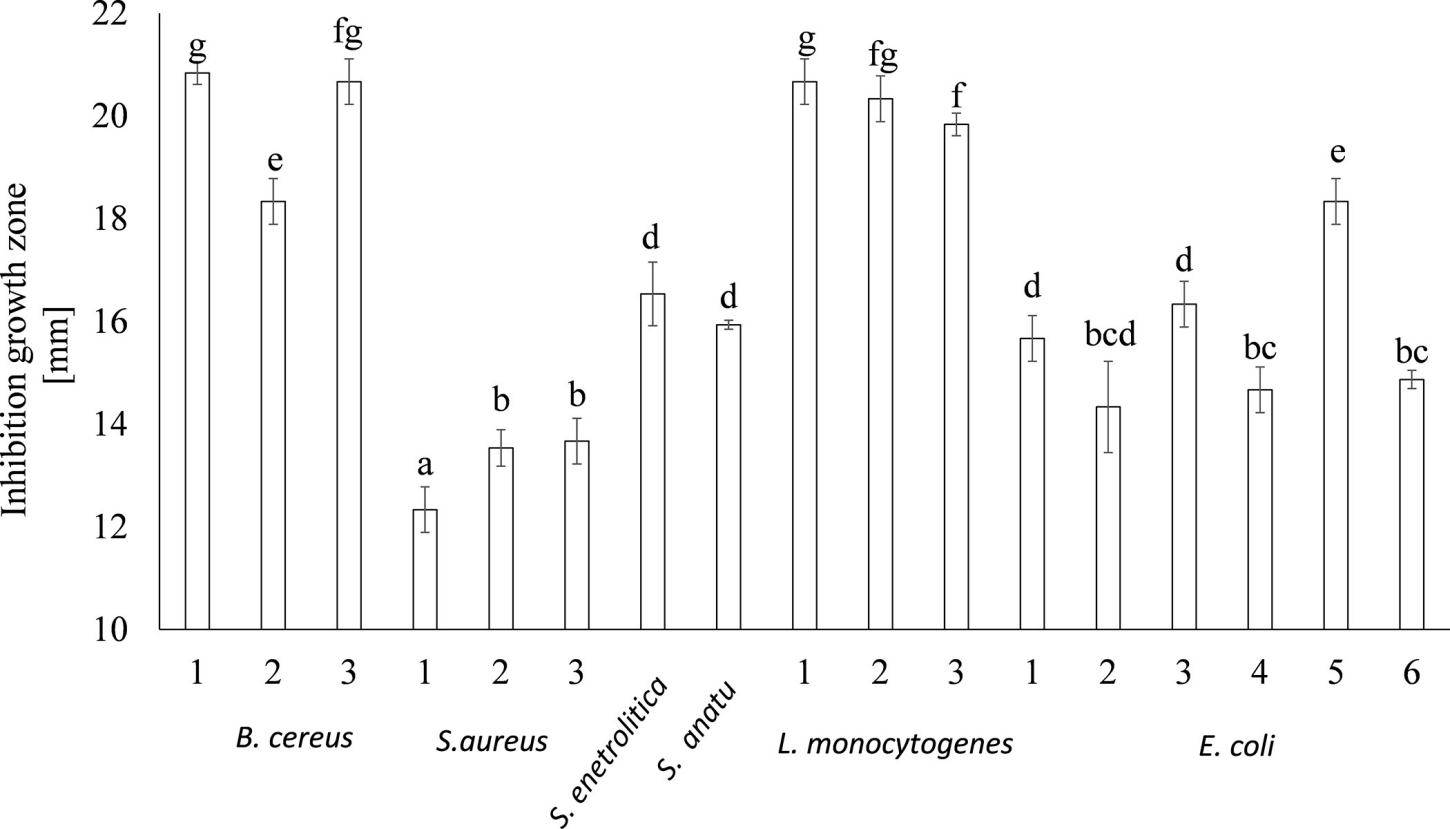
Antagonistic effect of *Lb*. *plantarum* against selected food-borne pathogens isolated from low-processed food. Means followed by different letters are significantly different (n = 9; p≤ 0.05).

The amount of aerobic mesophilic bacteria determined in the study was comparable with the results shown by Abadias, Usall, Anguera, Solsona, & Viñas [37]. In this study, sprouts were analyzed in 2005–2006. The bacterial count ranged between 7 and 8 log CFU/g in about 60% of the samples and was higher than 8 log CFU/g in 40%. On the other hand, the total microbial counts determined for lentils and mung sprouts obtained in an automatic sprouting system by Pao, Khalid, & Kalantari [38] were lower, i.e. 7.5 and 6.9 log CFU/g, respectively. There were smaller numbers of yeasts and molds than the count of bacteria, but in this study their amount was significantly lower than those detected (5.2 log CFU/g) in 15 different sprouts samples [37]. The concentrations of presumptive *B*. *cereus* in the lentil and mung bean sprouts detected in this study was comparable with those reported in the study by Pao et al. [38]. Similar to the other studies, the sprouts in this study were free from pathogenic microorganisms, e.g., *L*. *monocytogenes* or *Y*. *enterocolitica* [39], [40]. Generally, the microbiota of sprouted food is mainly influenced by organisms originally present inside and on seeds used for germination [41]. Seeds are usually disinfected but reduction in epiphytic microflora may result in an increase in the potential growth of pathogens [19]. In this study, *Lb*. *plantarum* replaced the natural microflora of sprouts and thus reduced the growth of the bacteria. Lactic acid bacteria naturally occur in sprouts but their count is usually less than 5 log CFU/g [37]. As observed in this study, the LAB count in the sprouts enriched with the probiotic ranged from 7.61 log CFU/g to 9.68 log CFU/g. Most importantly, an increase in LAB during storage was observed, which may suggest that sprouts provide important nutrients (free sugars and amino acids) [42], and sufficient water in the matrix [43]. A key role in this phenomenon is probably played by ecological competition. On the other hand antagonistic action of *Lb*. *plantarum* is well described and involves also production of bacteriocins and hydrogen peroxide [11]. To support this hypothesis, the antagonistic properties of *Lb*. *plantarum* 299V were determined against food-borne pathogens isolated from low-processed food (Fig 2).

*Lb*. *plantarum* 299V showed high antagonistic activity toward *L*. *monocytogenes* and *B*. *cereus* strains; they were also able to reduce the growth of studied *E*. *coli* and *Salmonella* spp. The studied strain was less effective toward *S*. *aureus*. The potential of *Lb*. *plantarum* to prevent spoilage of dairy products, fresh fruits, and vegetables was previously reported by Ołdak et al. [11] and Trias, Bañeras, Montesinos, & Badosa [44].

The antagonistic effect of LAB against *L*. *monocytogenes* in sprout cultivation was studied by Palmai & Buchanan [36]. When *Lactococcus lactis* was coinoculated onto seeds at the beginning of the sprouting process, the maximum levels of *L*. *monocytogenes* were approximately 1 log lower than those observed in the control cultures.

**Fig 3:**
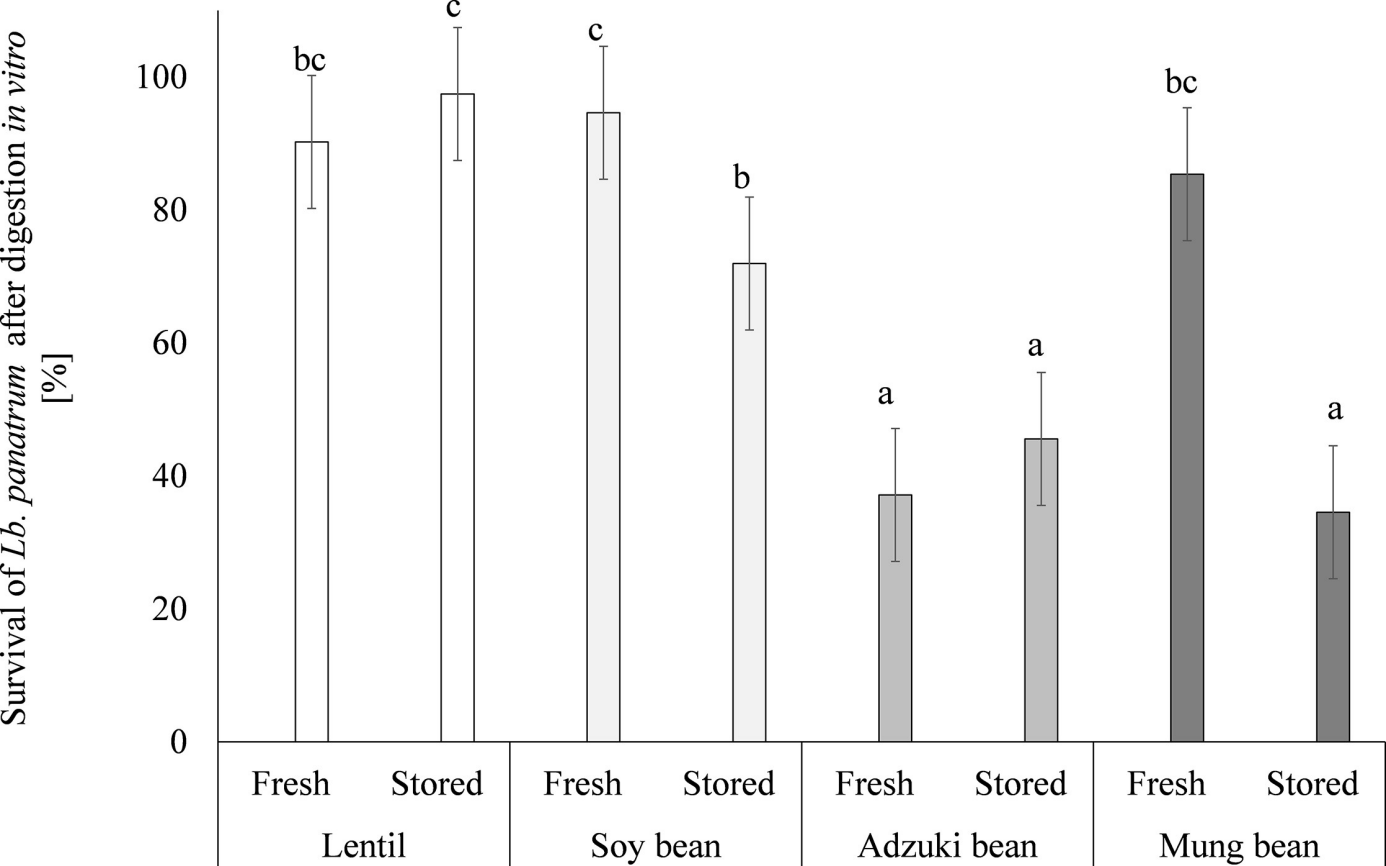
Survival of lactic bacteria during *in vitro* digestion. Means followed by different letters are significantly different (n = 9; p≤ 0.05).

The probiotic *Lb*. *plantarum* 299V strain is able to grow effectively in legume sprouts, which also ensures their high microbiological quality. To check if such formulations meet the requirements set for probiotics, bacterial survival during simulated *in vitro* digestion was studied (Fig 3).

The results showed a percent change in the probiotic count in the fluids after the process. The best protective properties were exhibited by the lentil sprouts, where the reduction of the initial population of LAB in the fresh and stored sprouts did not exceed 10%. A similar observation was found for the fresh soybean and mung bean sprouts; however, in the stored probiotic-rich sprouts, there was a decrease up to about 50% in the mung bean sprouts. In the case of the preparations based on adzuki bean, except for the high initial count of LAB, their content in the fluids after digestion was significantly decreased, i.e. by 63% and 54% in the fresh and stored probiotic-rich sprouts, respectively.

Many different alternative matrices have been studied as probiotic carriers but only some studies determined the effect of the digestive tract conditions on the final probiotic count. Generally, *Lb*. *plantarum* is resistant to mucin, low gastric pH, and bile salts [3]. In the study of Emser et al. [6], after a quick digestion of apple cubes dehydrated in a sucrose solution there was a slight decrease in *Lb*. *plantarum*, with log reductions below 1 log unit. The results obtained in this study for the lentil-based preparations are comparable with those obtained for microencapsulated *Lb*. *plantarum*, where a cell reduction of about 1 log unit was observed after digestion [45]. It is suggested that legume sugars are able to make niches that limit harmful conditions and/or free sugars change the osmotic potential locally, as reported previously [45].

## Conclusions

In conclusion, legume sprouts and *Lb*. *plantarum* 299V can be successfully combined to form a new probiotic-rich food. The key factors influencing the production of probiotic-rich sprouts include the temperature of sprouting (25°C) and methods of inoculation (soaking seeds in a water suspension of probiotics). Most importantly, *Lb*. *plantarum* 299V significantly improved the microbiological quality of sprouts, replacing natural endophytes with lactic bacteria. The population of *Lb*. *plantarum* 299V was also stable during cold storage. The high count of lactic bacteria observed in the digest confirmed the fact that studied sprouts are effective carriers for probiotics and ensure their survival in harmful conditions of the digestive tract. Enrichment of legume sprouts with probiotics is a successful attempt and yields products for a new branch of functional foods.
